# Efficacy and safety of Jinhua Qinggan granules for coronavirus disease 2019 (COVID-19): A protocol of a systematic review and meta-analysis

**DOI:** 10.1097/MD.0000000000020612

**Published:** 2020-06-12

**Authors:** Hao Chen, Yan-Ping Song, Kai Gao, Lin-Tao Zhao, Li Ma

**Affiliations:** a Pharmacy College, Shaanxi University of Chinese Medicine, Xianyang; b Shaanxi Academy of Traditional Chinese Medicine, Xi’an, Shaanxi, China.

**Keywords:** COVID-19, Jinhua Qinggan granules, protocol, systematic review

## Abstract

**Background:**

Coronavirus disease 2019 (COVID-19) is caused by severe acute respiratory syndrome (SARS)-COV2 and represents the causative agent of a potentially fatal disease. Jinhua Qinggan granules has definite effect in treating COVID-19 patients, but it has not been systematically evaluated for efficacy and safety.

**Methods:**

Retrieved the database, including the China National Knowledge Infrastructure (CNKI), Chinese Biomedical literature Database (CBM), Chinese Scientific and Journal Database (VIP), Wan Fang database, PubMed, and EMBASE. Evaluate methodological quality and judge risk of bias through the Cochrane manual. RevMan 5.3 and STATA 16.0 software were used to perform the meta-analysis.

**Results:**

This study will provide high-quality evidence of Jinhua Qinggan granules for COVID-19.

**Conclusion:**

The conclusion of this study will provide evidence to determine whether Jinhua Qinggan granules is an effective treatment for COVID-19.

**PROSPERO Registration Number:**

CRD42020182373.

## 1 Introduction

In December 2019, a series of unexplained pneumonia cases occurred in Wuhan, Hubei Province, China.^[1]^ A new coronavirus was identified as the pathogen, which was subsequently called Coronavirus disease 2019 (COVID-19) by the World Health Organization (WHO).^[2]^ Probably highly related to severe acute respiratory syndrome (SARS) and Middle East respiratory syndrome (MERS), COVID-19 is caused by a coronavirus SARS-CoV-2 and the ability to spread between people is extremely strong, which infects the lower respiratory tract and cause fever, cough, gastrointestinal infections and other symptoms.^[2,3]^ As of April 26, 2020, 2,922,737 cases of COVID-19 have been confirmed, 960,896 of which are confirmed to United States. Over 200,000 deaths worldwide. COVID-19 poses a huge threat to the public health and economy of humans around the world. Although most patients are mild, it is imperative to find suitable treatment drugs.

Chinese medicine has a long history and there are many clinical practices show that Chinese medicine has a significant effect in the intervention of COVID-19, which brings new hope for the prevention and control of COVID-19.^[4,5]^ Jinhua Qinggan granules was recommended as the treatment of patients in the medical observation period in the 7th edition of the "Diagnosis and Treatment Scheme for New Coronavirus Infected Pneumonia.”^[6]^ During the 2009 H1N1 flu, studies have shown that the combined treatment of Jinhua Qinggan granules and oseltamivir can shorten the fever time.^[7]^ Jinhua Qinggan granules have a significant effect on the treatment of mild, common COVID-19, reflected in the reduction of fever time, improvement of symptoms and reduction of inflammation.^[8]^ Based on the lack of comprehensive systematic evidence, we provided a protocol of systematic review for the treatment of COVID-19 by Jinhua Qinggan granules.

## 2 Methods and program

The protocol has been registered on International prospective register of systematic reviews (PROSPERO), the registration number is CRD42020182373. The protocol followed Preferred Reporting Items for Systematic review and Meta-Analysis Protocols (PRISMA-P) guidelines.^[9]^

### 2.1 Literature retrieval strategy

**Figure 1:**
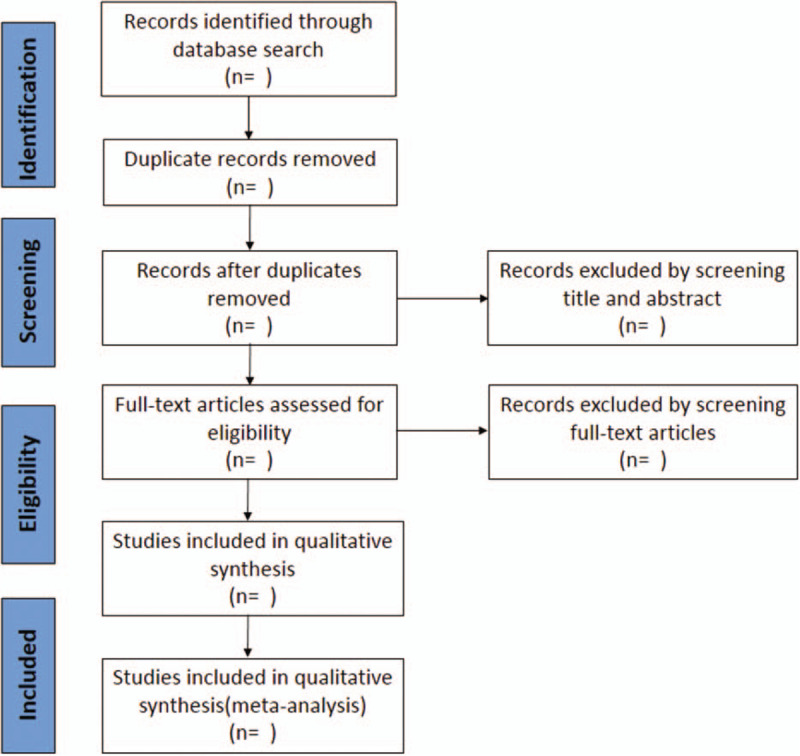
Flow chart for research selection.

Comprehensive search of China National Knowledge Infrastructure (CNKI), Chinese Biomedical literature Database (CBM), Chinese Scientific and Journal Database (VIP), Wan Fang database, PubMed and EMBASE. Language is limited to Chinese or English. Keywords include: “COVID-19,” “2019-nCOV,” “coronavirus disease 2019,” “novel coronavirus,” “Jinhuaqinggan,” “Jinhua Qinggan granules.” This work will be done independently by 2 researchers, and the third researcher will resolve the differences. Also, we will retrieve gray literature and the references cited in the study to avoid missing relevant qualified literature. We will contact the study authors if the article has some data missing or unclear that cannot be extracted. The screening process is shown in PRISMA flow diagram (Fig. 1).

### 2.2 Inclusion criteria

1. Prospective randomized controlled trial of all Jinhua Qinggan granules in the treatment of COVID-19.
2. Meets the diagnostic criteria of COVID-19 mild patients issued by the National Health and Health Commission of the People's Republic of China on the diagnosis and treatment of new coronavirus infection pneumonia (trial version 7), for example, real-time fluorescence quantitative PCR test positive for SARS-CoV-2, the whole genome sequencing of the virus showed high homology with the known new coronavirus or the specific IgM and IgG antibody against SARS-COV-2 were positive in serum test. Regardless of age or gender.
3. Intervention group: the treatment group was given Jinhua Qinggan granules on the basis of conventional treatment. Control group: only routine treatment. Routine treatment includes symptomatic treatments such as antiviral and antiinfection, for example, oral lopinavir, ritonavir, chloroquine phosphate, etc.
4. Main outcomes: Total efficiency, fever disappearance time, cough disappearance time, mortality rate. Additional outcomes: tumor necrosis factor-α (TNF-α), white blood cell count, incidence of adverse events.

### 2.3 Exclusion criteria

1. The experiment was not a randomized controlled trial.
2. Diagnosed as common, severe or critically ill.
3. Literature that does not conform to the research, such as review, animal experiment, and conference notice.
4. Respiratory symptoms caused by chronic respiratory disease, bronchial asthma, sinusitis, otitis media.

### 2.4 Data extraction

First of all, it is necessary for the 2 researchers to screen the topics and abstracts of the literature independently. After the screening, the literature is screened twice by reading the full text, and then the screening results are cross-checked. For each study, the name of the first author, year of publication, sample size, gender, age, details of treatment and control procedures, main results, and outcome indicators were recorded. If there is a disagreement, it is decided whether to include it or not through joint discussion, and a third researcher can assist in resolving it if necessary.

### 2.5 Quality assessment

As recommended in the bias risk assessment tool included in the Cochrane Handbook for Systematic Reviews of Interventions, this meta-analysis used Review Manager 5.3 software to perform quality assessment. It was evaluated from random sequence generation, allocation concealment, blinding of participants and personnel, blinding of outcome assessment, incomplete outcome data, selective reporting, and other biases and divided into 3 indexes: "high risk,” "unclear risk,” and "low risk.”

### 2.6 Data analysis

STATA 16.0 and Review Manager 5.3 statistical software were used for analysis. Outcome measures such as the clinical efficacy and adverse reactions were regarded as dichotomous variables and presented as the risk ratio with 95% confidence intervals (95%). TNF-α and white blood cell count was continuous variable that was presented as the mean difference with 95% CI. *Q* statistic and *I*^2^ tests were applied to assess the heterogeneity among studies. If *P* > .10 and *I*^2^ ≤ 50%, the study was homogeneous, using a fixed-effects model for statistical analysis. And a random-effects model was used to analyze data with heterogeneity (*P* ≤ .01 or *I*^2^ > 50%), and the effective results are statistically significant at *P* < .05. And the causes of heterogeneity were studied by subgroup analysis. Potential publication bias was revealed by funnel plot and Egger tests.

## 3 Discussion

Currently, there are no clear and effective drugs for patients with mild COVID-19. The largest number of such patients is the key to the current global epidemic prevention and control work. Finding effective treatment measures for such patients is one of the important tasks of clinical and scientific research.^[10]^ At present, Western medicine treatment mainly focuses on symptomatic treatment, and Chinese medicine intervention also plays an important role as a treatment.^[11]^ Studies have shown that based on historical records and human evidence for the prevention of SARS and H1N1 influenza, Chinese herbal formulas can be used as an alternative method for preventing COVID-19 in high-risk groups.^[12]^ Jinhua Qinggan granules have been used as an adjuvant therapy for COVID-19 in clinical practice.^[8]^ Therefore, it is imperative to use this research protocol to explore the efficacy and safety of Jinhua Qinggan granules on COVID-19.

## Author contributions

**Methodology:** Hao Chen.

**Project administration:** Hao Chen.

**Software:** Kai Gao and Lin-Tao Zhao.

**Supervision:** Yan-Ping Song.

**Visualization:** Li Ma and Lin-Tao Zhao.

**Writing – original draft:** Hao Chen

**Writing – review & editing:** Kai Gao

## References

[1] Lu H Stratton CW Tang YW Outbreak of pneumonia of unknown etiology in Wuhan, China: The mystery and the miracle. J Med Virol 2020;92:401–2. 31950516 10.1002/jmv.25678 PMC7166628

[2] Sohrabi C Alsafi Z O’Neill N World Health Organization declares global emergency: a review of the 2019 novel coronavirus (COVID-19). Int J Surg 2020;76:71–6. 32112977 10.1016/j.ijsu.2020.02.034 PMC7105032

[3] Shanmugaraj B Siriwattananon K Wangkanont K Perspectives on monoclonal antibody therapy as potential therapeutic intervention for Coronavirus disease-19 (COVID-19). Asian Pac J Allergy Immunol 2020;38:10–8. 32134278 10.12932/AP-200220-0773

[4] Ren JL Zhang AH Wang XJ Traditional Chinese medicine for COVID-19 treatment. Pharmacol Res 2020;155:104743. 32145402 10.1016/j.phrs.2020.104743 PMC7128263

[5] Yang Y Islam MS Wang J Traditional Chinese medicine in the treatment of patients infected with 2019-new coronavirus (SARS-CoV-2): a review and perspective. Int J Biol Sci 2020;16:1708–17. 32226288 10.7150/ijbs.45538 PMC7098036

[6] Ding YQ Bian XW Interpretation of pathological changes for “Guidelines for the Diagnosis and Treatment of COVID-19 by the National Health Commission (Trial Version 7)”. Zhonghua Bing Li Xue Za Zhi = Chin J Pathol 2020;49:E011. 10.3760/cma.j.cn112151-20200318-00221 32213267

[7] Wang C Cao B Liu QQ Oseltamivir compared with the Chinese traditional therapy maxingshigan-yinqiaosan in the treatment of H1N1 influenza: a randomized trial. Ann Intern Med 2011;155:217–25. 21844547 10.7326/0003-4819-155-4-201108160-00005

[8] Duan C, Xia WG, Zheng CJ, et al. Clinical observation of Jinhua Qinggan granule in treating new coronavirus infection pneumonia. *Chin Med J*. 2020 1–5.

[9] Shamseer L Moher D Clarke M Preferred reporting items for systematic review and meta-analysis protocols (PRISMA-P) 2015: elaboration and explanation. BMJ (Clinical research ed) 2015;350:g7647. 10.1136/bmj.g7647 25555855

[10] Jin YH Cai L Cheng ZS A rapid advice guideline for the diagnosis and treatment of 2019 novel coronavirus (2019-nCoV) infected pneumonia (standard version). Mil Med Res 2020;7:4. 32029004 10.1186/s40779-020-0233-6 PMC7003341

[11] Wang Z Chen X Lu Y Clinical characteristics and therapeutic procedure for four cases with 2019 novel coronavirus pneumonia receiving combined Chinese and Western medicine treatment. Biosci Trends 2020;14:64–8. 32037389 10.5582/bst.2020.01030

[12] Luo H Tang QL Shang YX Can Chinese medicine be used for prevention of corona virus disease 2019 (COVID-19)? A review of historical classics, research evidence and current prevention programs. Chin J Integr Med 2020;26:243–50. 32065348 10.1007/s11655-020-3192-6 PMC7088641

